# Motivations, Tensions, and Ideals in Global Health: Moving From Aspirations to Action

**DOI:** 10.7759/cureus.81169

**Published:** 2025-03-25

**Authors:** Bilal Irfan, Abdallah Abu Shammala, Abdulwhhab Abu Alamrain

**Affiliations:** 1 Center for Bioethics, Harvard Medical School, Boston, USA; 2 Center for Surgery and Public Health, Brigham and Women's Hospital, Boston, USA; 3 Department of Neurology, University of Michigan Medical School, Ann Arbor, USA; 4 Department of Internal Medicine, European Gaza Hospital, Gaza, PSE; 5 Department of Orthopaedics, Al-Aqsa Martyrs Hospital, Deir al-Balah, PSE

**Keywords:** global health equity, health justice, political advocacy, right to health, social determinants of health

## Abstract

Global health is profoundly shaped by historical contexts, ethical frameworks, and political imperatives, originating in part from colonial dynamics that continue to influence contemporary practices and resource distribution. This editorial seeks to examine three primary normative motivations, humanitarianism, global health security, and social justice, to assess their implications in addressing global health inequities. Humanitarianism, rooted in compassion and charity, has historically aided in facilitating important interventions but risks reinforcing hierarchical donor-recipient dynamics and neglecting local expertise. A security-based rationale, driven by concerns over pandemic threats, galvanizes rapid resource mobilization yet may also perpetuate global disparities by prioritizing affluent regions. In contrast, a social justice framework advocates for systemic equity, emphasizing local leadership, mutual respect, and the dismantling of colonial legacies. Critically evaluating these motivations is important as some argue that prioritizing social justice is essential for genuine decolonization and sustainable global health interventions. Furthermore, this piece explores how aspirational ideals, despite appearing impractical, can inspire meaningful reform, accountability, and the creation of inclusive coalitions. By balancing realism with ambitious ethical standards, global health can move beyond aspirational rhetoric to actionable strategies that genuinely address structural inequities.

## Editorial

Global health, as a field of study, a distinct discipline, and an area of practice, has some of its origins in an era of colonialism and racially driven power hierarchies that continue to influence decision-making and the allocation of resources [1,2]. As scholars and practitioners within academic medical centers and global health institutions grapple with past legacies and the existing present forms of inequity across the world, the normative motivations behind their work have come into sharper focus [3-5]. Many leading actors once derived their influence from colonial structures or from a sense of charitable paternalism [1]. However, this lens is under greater scrutiny as there are renewed and growing calls to decolonize and reimagine the field [2]. In such a context, understanding the normative motivations that prompt engagement in global health is vital to devising meaningful and sustainable interventions [3]. There are various normative motivations for participating in global health, three of which will be assessed for their role in shaping policy and setting priorities, and analyzing the tension that often emerges between the pursuit of global health security and global health equity [1]. In doing so, one may be able to propose which such motivations ought to drive global health if we hope to repair the structural inequities of the field. Furthermore, we can consider what is gained or lost by striving for ambitious moral and political ideals in an increasingly fractured and divided world.

One normative motivation that has historically driven global health interventions is a sense of humanitarianism, often expressed through the language of compassion or moral duty [1,4]. This stems from religious or spiritual frameworks for some people, ideas that call for service to the poor and the ill; for others, it emerges from secular-derived or personal moral intuitions regarding the imperative to alleviate suffering [5]. While it remains undeniable that compassion can lead to worthwhile progress, its manifestation in practice is somewhat mixed [3]. The philanthropic or charitable impetus has, in some instances, served to further cement hierarchies between donors and beneficiaries [5]. Even the modern philanthropic architecture, as manifested in bilateral or multilateral aid, is often rooted in the notion that wealthier nations, many of which historically gained their status via processes of exploitation, now provide resources to what are classified by some as poor states, who are thus presumed to be unable to handle their crises alone [1]. A criticism that has been lodged is that such philanthropic motivations risk reinforcing a colonial discourse in which there is a Global North or Western world that is a savior, leaving certain local voices and expertise undervalued. That is not to entirely disregard the value of short-term philanthropic aid, which has been indispensable for delivering life-saving services, such as vaccination campaigns or surgical outreach. Humanitarian motivation, therefore, has practical benefits, but it can be complemented by an informed sense of humility and an awareness of longstanding power inequities.

A second motivation that drives involvement in global health arises from the vantage point of basic self-interest. While self-interest can take many forms depending on the actor involved, the analysis will be focused on that arising from the desire to preserve global health security [3,5]. States and international bodies are keen to prevent the cross-border spread of pandemics or to protect their populations from threats. Recent infectious disease outbreaks, such as that of Ebola or COVID-19, served as further reminders of how pathogens pay little regard to national borders, which has aided in galvanizing a security-based rationale for global health engagement [3]. As national policymakers and certain global institutions calibrate their actions around security concerns, funds in some quarters are more readily allocated toward interventions that safeguard wealthier nations from potential spillover threats [3]. COVID-19 vaccine hoarding was commonplace and at times coupled with insufficient efforts to maintain solidarity with low-income countries [5]. These revealed inherent tensions with global calls for health equity. The impetus here in this motivation of self-interest is a notion of mutual protection, with the implication that stronger health systems everywhere protect everyone. Although the security perspective may mobilize rapid funding or political commitment, it risks overshadowing attention to deep health disparities [2,3]. Many high-income nations invest chiefly in issues that threaten them, marginalizing local priority areas in less powerful settings. The risk is that this security-based motivation entrenches some of the same colonial hierarchies that decolonizing voices seek to uproot, and thereby prevents a genuinely cooperative model that addresses health concerns in various regions locally.

A third normative impetus that can be readily identified is a social justice framework, an orientation that emerges from critiques of the moral duty approach and from dissatisfaction with the narrow global health security rationale [1,3]. Social justice proponents at times postulate that every individual, regardless of where they live, has a right to health and that global health should focus on systematically dismantling inequities that have deep historical roots. This approach insists that the old patterns of knowledge extraction and paternalistic aid must be replaced by genuine partnership models, capacity co-creation, and, ultimately, by addressing the structural causes of poverty and disease [1,2]. Global health scholars and activists argue that colonial legacies and systemic racism not only hamper the voices of local communities but also actively perpetuate cycles of dependency and underdevelopment [2]. Social justice as a motivation thus calls on practitioners to redress these inequities, to recognize local knowledge and leadership, and to avoid labels like low-income, developing, or Global South as unidimensional descriptors [2,4]. A social justice framework requires an understanding that each context is unique, potentially shaped by colonialism, economic exploitation, structural violence, or other factors. If realized, a justice-focused paradigm may be able to systematically confront the imbalance of power, accelerate universal access to quality care, and shift the narrative away from vertical charity or paternalism and instead gravitate toward reciprocity and respect.

Despite the distinct normative underpinnings of humanitarianism, security, and social justice, their practical implementations often blend together in complex ways that shape power structures on the ground. For example, during the 2014-2016 Ebola outbreak in West Africa, a strong global health security rationale mobilized funding and personnel, aiming to contain the threat before it reached wealthier regions. While this rapid response did help limit regional and global spread, some local communities criticized how the urgency of containment overshadowed capacity-building measures, sidelining local expertise in favor of external directives. At the same time, several humanitarian organizations mounted large-scale relief operations, guided perhaps by compassion or a sense of moral duty. However, they, too, frequently imported top-down interventions that left community leaders feeling marginalized. A comprehensive social justice lens, which would have placed local voices at the forefront, was slow to materialize, even though many civil society groups in West Africa were advocating for deeper community engagement. This example is one that goes to show how security and humanitarian approaches can unintentionally reinforce hierarchical donor-recipient relationships when insufficient attention is paid to structural reform and participatory governance.

Local agency and participatory governance are, in fact, cross-cutting elements that can mitigate some of the structural constraints of both humanitarian and security-based interventions. Genuine decolonization, in this sense, requires active reconfiguration of governance models, funding flows, and knowledge production so that communities are not merely passive recipients of aid or surveillance but co-architects of interventions. Under humanitarian paradigms, this means moving beyond charitable one-way giving and designing programs in full partnership with local institutions to ensure that solutions are culturally relevant and sustainable. Under security rationales, it entails rethinking how priorities are set so that frontline voices help define the scope of preparedness and response strategies, rather than having external stakeholders focus narrowly on preventing a potential spillover threat. In both cases, acknowledging local leadership as essential, rather than optional, can aid in aligning a broader social justice imperative to dismantle colonial legacies of paternalism.

These evolving real-world experiences also show that aspirational ideals are not so much separate from the three motivations as they are a constant moral horizon that can reshape each framework from within. By framing humanitarian aid in a language of equity and sovereignty rather than pity or charity, actors can foreground long-term development and partnership. By anchoring security concerns in inclusive deliberation, global health agencies can invest in resilient local health systems that withstand future shocks and thus fulfill the criteria of protecting all populations without entrenching inequalities. Additionally, by insisting on a social justice ethos, practitioners move closer to the ethos of genuine decolonization, which is grounded in reimagining how decisions, resources, and knowledge are produced and shared. In each case, the aspiration of a more equitable global health landscape serves to hold institutions accountable, driving them to adapt their motivations and practices to align better with the lived realities of communities worldwide.

These three normative motivations, when taken together, be it a humanitarian impulse, security-based thinking, or a social justice framework, can aid in shaping global health priorities. Tensions do indeed arise between the goals of advancing equity and of protecting populations from perceived external threats. One sees this playing out quite prominently during pandemics and epidemic-like situations, such as in cases where governments with a greater degree of financial cash wealth may restrict exports of medical supplies or oppose waivers for intellectual property rules on vaccines. Rather than coordinating in an international forum in an equitable manner, some states or regional actors focus on ring-fencing their own supplies and argue that controlling the spread at their own borders must take precedence. The impetus for equity, meanwhile, and in contrast, insists on widespread, cost-free, or affordable access to interventions and capacity for all countries. In reality, this tension between equity and security becomes starkly evident and deeply entrenched as security-based motivation can lead to short-term, problem-specific, and top-down solutions that do not address the root causes of health disparities or build sustainable systems. The expansion of robust local capacity can be hindered in a security-driven model, as that approach typically invests resources only to the extent that is necessary to prevent external shocks from traveling beyond certain borders. This tension reveals how, even when motivations may appear benign, they can continue colonial habits of control and extraction under new guises.

Political advocacy often operates by building broad coalitions, of patients, health workers, and civil society organizations, that press governments, private corporations, and international bodies to expand health services. To address the inequalities present in a society, political activism aims to reshape power dynamics. Rather than health being a commodity, activists argue that governments and industries should recognize healthcare as a right and, therefore, ensure equitable distribution. In doing such, and by mobilizing those most affected, it is arguable that activism fosters participatory processes that highlight the systemic injustices causing ill health. In parts of the AIDS movement, for example, mobilized patient groups and global health activists demanded affordable antiretroviral therapy (ART) in resource-poor countries. They identified a moral claim to treatment embedded in the broader right to health discourse. By forming organizations such as the Treatment Action Campaign and working in solidarity across borders, activists helped reduce drug prices, shape legislation, and expand AIDS treatment programs. This approach aimed at directly tackling financial and structural barriers that historically kept low-income communities from obtaining life-saving therapy.

It is worth underscoring that political advocacy can also meaningfully advance justice by revealing how inequalities stem from policy choices, profit-driven health systems, and neglect of marginalized populations. Campaigns for comprehensive AIDS treatment, for example, lowered drug costs worldwide and pressured international bodies to fund health programs. These gains expanded access for impoverished communities, showcasing a direct move toward a notion of fair equality of opportunity, in which all individuals should be able to develop their capabilities without medically preventable constraints. However, advocacy may fail if it does not sustain policy changes or if it lacks sufficient political leverage to enforce reforms at scale. Large pharmaceutical companies have sometimes offered only partial or temporary price reductions. Governments can also reverse funding commitments when political will wanes, revealing the fragility in the process itself. Advocacy alone may not restructure all exploitative features of the health system, such as private profit from life-saving treatments. In such cases, it is clear that activism can founder if it is not coupled with broader changes in social and economic arrangements.

It can be argued that health services are of a special moral importance because they protect individuals’ normal functioning, thereby securing fair equality of opportunity in society. Political activism effectively appropriates this Rawlsian rationale by asserting that healthcare is not merely a market good but a public good essential for social and economic participation. Activists have emphasized that structural determinants, such as poverty, discrimination, and unequal financing, undermine the principle of fair equality of opportunity. Some activists also align with a call for fair processes, or the accountability for reasonableness, to guide priority-setting in resource constraints. By mobilizing marginalized communities, advocacy groups can provide transparent and equitable deliberation around who receives treatment or how resources are distributed. In some respects, the political advocacy approach mirrors an emphasis on inclusive, reasoned debates over healthcare decisions, particularly when it pushes for the voices of the poor and sick to be heard in public policymaking.

Political activism also broadens the scope of bioethics as a field of study and discipline. While large strains of contemporary Western bioethics often address clinical dilemmas such as informed consent or end-of-life care, activism spotlights structural and justice-oriented issues. It calls attention to the ways in which market-driven systems, patents, and profit motives can deepen health inequities. This emphasis on the public’s role in demanding accountability and by reorienting bioethics to concern itself with systemic injustice and a people-centered process, political advocacy moves the field toward what some term a social bioethics. In doing such, bioethicists have the ability to concretely support the activism approach by researching the moral arguments behind equal access, assembling empirical evidence on health disparities, and participating in public debates. Such engagement encourages a bridging of academia, clinical practice, and grassroots organizing, so that normative ethics does not remain purely theoretical. Instead, it effectively becomes a tool for addressing social determinants of health. Advocacy-based activism deepens bioethics’ social critique and also provides a framework for real-world interventions, with some parallels to theories tying health to justice and the movement’s insistence that treatment be universal and non-negotiable. Activism can simultaneously be a tool to challenge bioethics to refine its vision of autonomy and justice so that it includes collective dimensions. Just as the right to health cannot be fulfilled in isolation from politics, so bioethics must expand beyond “patient-doctor” dyads to grapple with policy, globalization, and mass movements for reform. In many senses, political advocacy offers new directions for bioethics, pressing it to incorporate structural critique and champion collective rights.

Mobilizing the right to health through political activism has helped address health inequalities by exposing how profit, power imbalances, and discriminatory policies perpetuate harmful conditions. Through alliances across civil society, advocates can secure lower drug prices, enact supportive legislation, and alter resource allocation patterns. Drawing from the arguments from various bioethicists and global health scholars, activists’ claims for fair equality of opportunity reinforce the notion that health is intrinsically tied to justice. Although such reforms can be fragile and partial, advocacy has the power to shift norms, hold institutions accountable, and invite bioethics to become a more engaged, justice-focused enterprise. By transforming abstract moral principles into collective action, political activism advances the project of realizing health as a human right.

Upon reflecting on these motivations as outlined above, the question rightly becomes regarding which ones ought we to prioritize if we strive for a more just, cohesive, and truly global approach to health. Some may argue the social justice approach is the most consistent with the decolonization of global health, as it demands that practitioners examine the power and knowledge asymmetries at play, and that we rectify them through institutional reforms, inclusive governance structures, and meaningful alliances with local communities. Pursuing social justice means recognizing that local knowledge systems can also be valid, that equity-based governance is indispensable, and that the impetus for solidarity must replace old paternalistic charity. It also means acknowledging that communities around the world can, and do, innovate, as they may hold historically tested solutions, or have adapted solutions from multiple traditions, to address their own challenges.

We must also consider what is gained or lost by adhering to such ideals, especially if they appear unattainable or unrealistic in a world that is shaped by inertia and self-interest. Global health has frequently functioned under short funding cycles, state-based alliances, and ephemeral political will, resulting in repeated cycles of treat and forget, aid and exit, or emergency response with limited structural effect. If we hold onto bold, justice-based norms that sometimes appear unreachable, there may be a risk of alienating potential allies. In such circumstances, perhaps there is an impetus to create new, robust movements that drive structural change? On the one hand, rhetorical commitment to high-minded ideals, such as universal health coverage or dismantling exploitative systems, can spur innovation, create impetus for activism, and unify a range of stakeholders behind a common cause [3]. On the other hand, adopting lofty ideals can make us vulnerable to disillusionment, as seen in repeated cycles of disappointment with high-level pledges at global summits, or with progressive-sounding proposals that never materialize in practice. For example, the United States’ withdrawal from the World Health Organization (WHO) under the Trump administration, or the new directives to fold the US Agency for International Development (USAID) into the State Department, can actually reduce trust and hamper multilateral approaches to health, as ongoing projects and reliability is broken, which thereby sabotages or at least complicates efforts to coordinate a justice-based or even a security-based approach.

Nevertheless, if we do not keep radical, justice-based ideals in our peripheral and central vision, we risk entrenching the status quo and perpetuating existing power hierarchies. The quest for universal ideals, albeit indeed quite seemingly remote from many of the daily political realities, does still provide a moral horizon that pushes individuals and institutions beyond short-term self-interest or ephemeral philanthropic instincts. Retaining the social justice impetus also fosters some sense or modus of accountability. An ideal, such as the right to health, is beneficial due to the moral weight it carries but also on account of the fact that it can help local communities demand accountability from their governments or global institutions in a particular articulable fashion. Considering this, even if the realpolitik of the moment strays from such ideals, their steady presence in our discourse and planning can propel transformations over the long haul.
